# Analysis of lifestyle factors associated with physical activity participation among university female students using deep belief networks

**DOI:** 10.3389/fpsyg.2026.1792556

**Published:** 2026-04-23

**Authors:** Jing Jia

**Affiliations:** Shanxi College of Applied Science and Technology, Taiyuan, Shanxi, China

**Keywords:** deep belief network (DBN), female university students, lifestyle factors, nonlinear modeling, physical activity, predictive modeling

## Abstract

Female university students often report lower levels of physical activity compared to their male counterparts. While prior research has examined psychological, sociocultural, and physiological factors associated with these differences, many quantitative approaches rely on linear models that may not capture nonlinear relationships. This study proposes a Deep Belief Network (DBN) framework to model associations between lifestyle factors and physical activity status among female university students. A sample of 1,032 female students was analyzed using four predictor variables: Stress Level, Study Hours, Sleep Hours, and Social Hours. These variables were preprocessed through encoding and Min–Max normalization, and physical activity was represented as a binary classification outcome. The DBN model was trained using unsupervised pre-training followed by supervised fine-tuning. It outperformed baseline models, achieving a mean accuracy of 92.4% (±1.2), F1-score of 91.3% (±1.5), and Area Under the Curve (AUC) of 0.96 (±0.01) across five-fold cross-validation. Bootstrap analysis on out-of-fold predictions yielded 95% confidence intervals of [90.1, 94.6%] for accuracy, [89.0, 93.5%] for F1-score, and [0.94, 0.98] for AUC. Latent feature analysis indicated that stress level showed the strongest association with model predictions, followed by social and physiological variables, while study hours showed a comparatively weaker association. Sensitivity analysis revealed nonlinear patterns, including a threshold effect for stress and a U-shaped association between social hours and physical activity. These findings demonstrate the ability of deep learning models to capture nonlinear associations within a limited set of observable lifestyle variables. However, the study does not directly measure psychological motivation constructs, and the results reflect predictive associations rather than causal relationships; therefore, causal inferences cannot be drawn from this analysis. Future research should validate these findings using longitudinal data to examine temporal dynamics and potential causal relationships. Incorporating validated psychological constructs and objective measurements, as well as evaluating the framework across diverse populations, may further improve interpretability and generalizability.

## 1 Introduction

Physical activity is an important contributor to physical health, mental well-being, and academic performance among university students (Zhou and Liu, 2025; Prieto-González and Alkouatli, 2025; Martínez-Sánchez et al., 2024). Despite this, levels of physical activity among female university students remain relatively low worldwide (Hasan et al., 2021; Aljehani et al., 2022; Sheng et al., 2023). In this study, the primary outcome is physical activity status, defined as whether a student engages in at least 30 min of daily physical activity.

Previous research has identified a range of factors associated with physical activity among women, including psychological, social, and environmental variables (Ni and Yu, 2023; Al Salim, 2023; Van Luchene and Delens, 2021; Rodenbaugh, 2016). Commonly reported factors include time constraints, body image concerns, perceived judgment, and self-efficacy (Agbabiaka et al., 2023; Williams et al., 2018; Zhang et al., 2024). In addition, variables such as stress, academic workload, social engagement, and access to resources have been associated with activity patterns in this population (Diehl et al., 2018; Kondric et al., 2013; Murray et al., 2021). Many of these relationships have been examined using statistical and machine learning approaches; however, a substantial proportion of studies rely on linear models that assume independence and linear relationships among variables (Pacheco et al., 2012; Li, 2025; Wu and Liu, 2022). However, relationships between lifestyle variables and physical activity may involve nonlinear patterns and interaction effects (Dong et al., 2024; Choe et al., 2025). In contrast to traditional approaches, Deep Belief Networks (DBNs) provide a flexible framework for capturing nonlinear relationships and potential interaction patterns among variables (Zhou et al., 2019; Cui and Yin, 2025). In this study, DBNs are applied to examine associations between selected lifestyle variables and physical activity status among female university students.

It is important to note that this study focuses on a limited set of observable lifestyle variables available in the dataset, namely stress level, study hours, sleep hours, and social hours. Accordingly, the scope of this study is restricted to examining nonlinear associations within this limited feature set, rather than representing a comprehensive behavioral system. To position this work within existing literature, a structured literature scan was conducted using databases including Google Scholar. Keywords such as “female university students,” “physical activity,” “machine learning,” and “deep learning” were used to identify relevant studies published between 2015 and 2026. Studies were included if they examined predictors of physical activity using quantitative or computational methods. The review indicates that while machine learning approaches have been applied to physical activity prediction (Sheng et al., 2025; Alecu et al., 2025; Kaggle, n.d.), relatively limited work has explored deep generative models using survey-based lifestyle variables in this population. Although interest in machine learning applications in sports and health research is increasing (Hinton et al., 2006; Hinton, 2012), much of the existing deep learning literature focuses on activity recognition using sensor or physiological data (Decelle and Furtlehner, 2021; Fisher, 2022). In contrast, fewer studies have examined lifestyle-based predictors derived from survey data, particularly among female university students (Bai et al., 2022; Sathyanarayanan and Tantri, 2024). This highlights an opportunity to explore the application of deep learning methods in this context.

In response, the present study proposes a DBN-based analytical framework to examine associations between lifestyle characteristics and physical activity status. The framework incorporates psychological, temporal, physiological, and social variables within a two-stage training process involving unsupervised RBM pre-training followed by supervised fine-tuning.

The contributions of this study are threefold. First, it applies a DBN framework to model associations between lifestyle variables and physical activity status. Second, it evaluates the predictive performance of the proposed approach relative to baseline machine learning models. Third, it provides an analysis of model-derived feature contributions to better understand patterns within the observed variables.

In summary, this study demonstrates the application of deep learning methods for modeling nonlinear associations among a limited set of lifestyle variables related to physical activity status in female university students. The findings contribute to the growing literature on computational approaches to physical activity research while remaining aligned with the scope and limitations of the available data.

## 2 Related works

### 2.1 Factors and motivation among female university students

Research on physical activity among university women has consistently shown that participation patterns are shaped by a complex interplay of psychological, sociocultural, environmental, and academic factors. A comprehensive narrative review has been conducted, drawing together evidence across countries and identifying key constraints, including low self-confidence, gendered expectations, limited accessibility to facilities, and increased academic pressure (Zhou and Liu, 2025). Their cross-cultural lens indicates that motivations and contextual factors for female college students are multidimensional and can differ based on social context. Empirical studies underline that these factors are often gendered. A comparative analysis of male and female university students has shown that women experience more psychological barriers, such as fear of judgment, stress, and difficulty balancing academic demands (Prieto-González and Alkouatli, 2025). Additionally, greater time constraints, body-image concerns, and health and safety issues have been identified among women, increasing the risk of non-participation (Martínez-Sánchez et al., 2024). Other studies focusing on female university students, including recent cross-sectional research, have identified several factors associated with physical activity participation, such as fatigue, low self-efficacy, and low perceived competence (Hasan et al., 2021; Aljehani et al., 2022; Sheng et al., 2023; Ni and Yu, 2023; Al Salim, 2023). In addition, psychological and social factors—particularly social support—are associated with participation patterns in this population (Van Luchene and Delens, 2021). Gendered environmental constraints associated with physical activity participation on university campuses have been identified (Rodenbaugh, 2016). Similarly, sociability and perceived accessibility to facilities are important factors influencing female students’ participation in recreational activities (Agbabiaka et al., 2023). Furthermore, race and minority status have been reported to be associated with differences in motivational and sociocultural factors affecting physical activity among female students (Williams et al., 2018). Taken together, this research indicates that certain predictors associated with participation status among university female students are unique and idiosyncratic, rather difficult to quantify and ameliorate with one-size-fits-all models of physical-activity behavior.

### 2.2 Women’s sports participation and motivation: broader evidence base

Research on women’s sport participation has widely applied motivational theories to explain behavior. Drawing on Self-Determination Theory, autonomy, competence, and relatedness have been highlighted as key drivers, while themes such as enjoyment, health, skill development, and affiliation have also been identified (Zhang et al., 2024; Diehl et al., 2018; Kondric et al., 2013). Studies on younger and collegiate populations further emphasize the role of social identity, perceived competence, personal growth, and team belonging in shaping participation (Murray et al., 2021; Pacheco et al., 2012). Collectively, these findings underscore the importance of multi-factor frameworks incorporating psychological, social, and contextual influences.

In parallel, machine learning (ML) approaches have increasingly been used to model physical activity behavior. However, many studies focus primarily on physiological or behavioral data, with limited integration of psychosocial variables. Deep learning models (Wu and Liu, 2022) have been applied to behavioral and health-related data, while traditional and ensemble models have been used to predict activity levels based on psychological factors (Dong et al., 2024; Choe et al., 2025). Other work has leveraged sensor data (Zhou et al., 2019) or applied Random Forest methods to identify and rank predictive factors for physical activity in college populations (Cui and Yin, 2025). Recent studies also demonstrate that constructs such as self-efficacy and gender differences can be incorporated into predictive models (Sheng et al., 2025; Alecu et al., 2025).

Despite these advances, existing approaches often fail to fully capture the complex, nonlinear interactions among psychosocial factors. In particular, the use of deep generative models such as Deep Belief Networks (DBNs) remains largely unexplored for modeling these relationships in the context of female students’ physical activity participation.

### 2.3 Research gaps and study contribution

Existing literature reveals several important limitations across factor-based and machine learning approaches to physical activity research among female university students. First, factor-based studies provide rich descriptive insights but lack predictive modeling frameworks capable of capturing interactions among variables. Second, many machine learning studies rely primarily on sensor or behavioral data, with limited incorporation of psychosocial and lifestyle-related factors, creating a disconnect between psychological theory and computational modeling. Third, there is a lack of research applying Deep Belief Networks (DBNs) to this population, particularly for modeling latent interactions among stress, time constraints, sleep, and social context. Finally, existing deep learning approaches often lack interpretability, providing limited insight into how learned features relate to theoretical behavioral constructs.

To address these gaps, the present study proposes a DBN-based framework that integrates behavioral theory with deep generative modeling. Lifestyle variables are explicitly mapped into conceptual categories (psychological, temporal, social, and physiological factors), enabling the model to learn latent representations and capture nonlinear relationships among participation-related factors. The study focuses specifically on female university students, addressing a demographic gap in prior research. Furthermore, interpretability is enhanced through analysis of hidden-layer activations, allowing identification of key factor interactions associated with physical activity participation. This integration of theoretical constructs with DBN modeling represents a novel contribution to sports science and behavioral analytics.

## 3 Methodology

**Figure 1:**
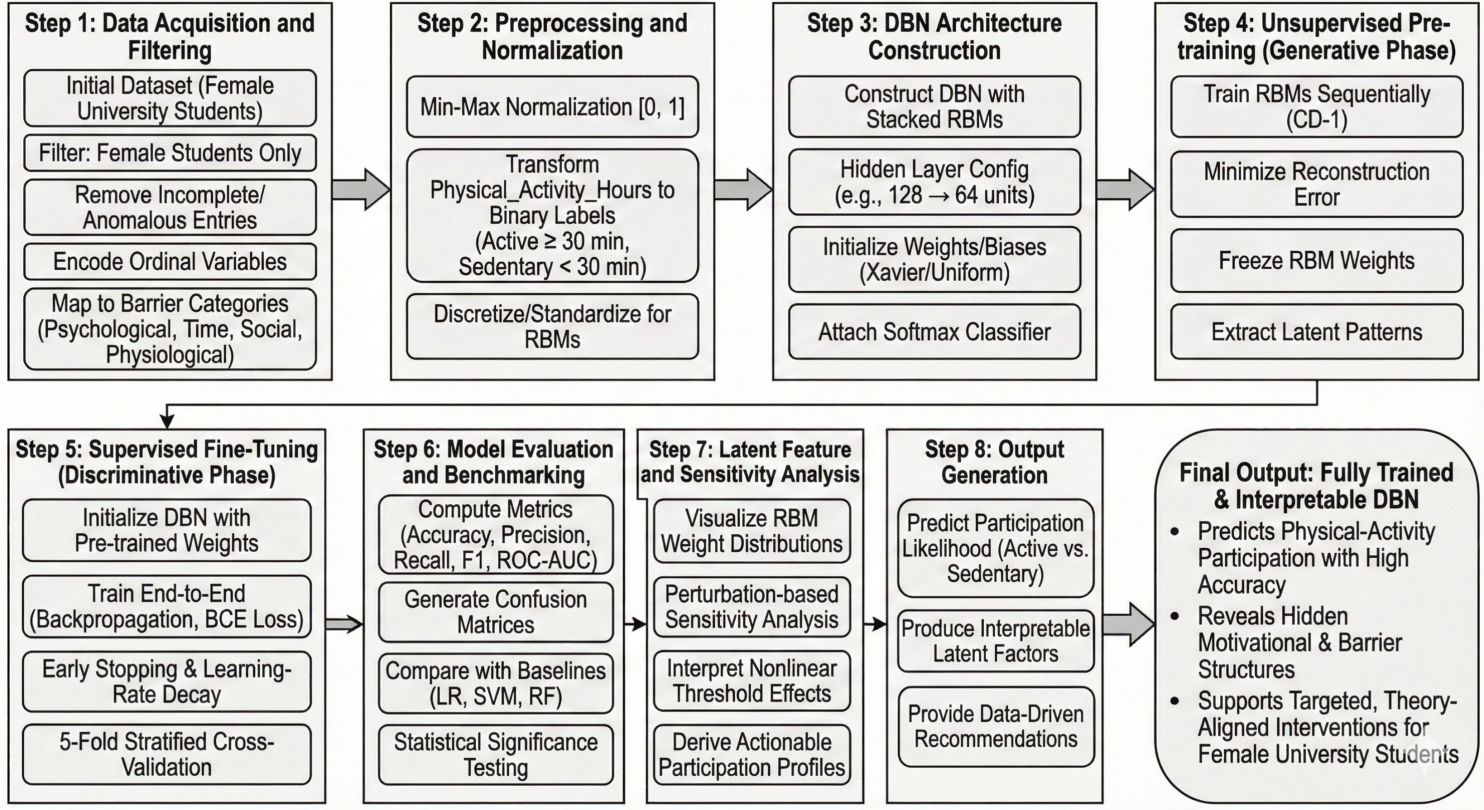
Overview of the proposed methodological pipeline, illustrating the flow from raw data acquisition and preprocessing through DBN construction, unsupervised RBM pre-training, supervised fine-tuning, and model evaluation using stratified 5-fold cross-validation.

This chapter outlines the computational tools we used to approach mapping the link from lifestyle factors to students’ physical activity participation. This is done using a DBN to mine hierarchical hidden features in student behavior. The overall flow is outlined in Figure 1.

The modeling pipeline comprised five stages. A female-specific dataset (*N* = 1,032) was extracted, and continuous variables were Min-Max normalized to [0, 1]; the target was binarized (active ≥30 min/day vs. sedentary). The DBN architecture used stacked RBMs (128 → 64 hidden units) initialized via Xavier/uniform sampling, with a sigmoid output layer trained using binary cross-entropy. Unsupervised pre-training with CD-1 was conducted layer-wise to capture latent, non-linear relationships among lifestyle variables. Learned weights were then transferred to a feedforward network and fine-tuned via backpropagation, with early stopping and learning rate decay applied to reduce overfitting and improve generalization.

Model performance was evaluated using stratified five-fold cross-validation to preserve class distribution. Standard metrics (Accuracy, Precision, Recall, F1-score, AUC) were reported, alongside confusion matrix analysis to assess false positives and false negatives. The DBN was benchmarked against Logistic Regression, SVM, and Random Forest, with McNemar’s test used to assess the statistical significance of performance differences. To improve interpretability, RBM weight distributions and perturbation-based sensitivity analysis were examined, revealing key nonlinear effects (e.g., a stress threshold ≈0.6 linked to inactivity risk) and enabling identification of behavioral profiles (e.g., high-stress/low-sleep groups). Finally, the model produces individual probabilities of being physically active or sedentary, supporting early identification of at-risk students and enabling targeted interventions. Overall, the framework provides a compact, interpretable, and robust approach for modeling lifestyle–physical activity relationships while maintaining strong predictive performance.

### 3.1 Data

This study used a publicly available dataset, “Lifestyle Factors and Their Impact on Students,” obtained from Kaggle (n.d.). The dataset is distributed under a CC0 (Public Domain) license and contains anonymized student lifestyle information. The original dataset included 2,000 observations and 9 variables. After filtering for female participants, the final analytical sample consisted of 1,032 observations. No additional exclusion criteria were applied. All selected variables contained complete data; therefore, no imputation or data removal was required. The analysis used four predictor variables—Stress Level, Study Hours, Sleep Hours, and Social Hours—and one target variable, Physical Activity (Table 1). Stress Level is an ordinal variable, while the remaining predictors are continuous. Physical Activity, originally recorded as hours per day, was converted into a binary classification variable as described in Section 3.2. These variables represent observable lifestyle characteristics and were used as inputs for the modeling process. The dataset reflects general student lifestyle behavior and is not specific to structured physical activity programs; therefore, results should be interpreted accordingly.

**Table 1:** Mapping of dataset variables to conceptual roles in the DBN model.

Feature type	Dataset variable	Conceptual role in DBN
Psychological factor	Stress level	Represents perceived mental load associated with activity behavior.
Time-related factor	Study hours	Represents academic time demands associated with activity patterns.
Social factor	Social hours	Represents social engagement, which may be associated with activity behavior in different ways.
Physiological factor	Sleep hours	Proxy for physical recovery and daily energy levels.
Target variable	Physical activity	Binary outcome variable representing activity status.

### 3.2 Data preprocessing

The preprocessing pipeline included target discretization, feature encoding, normalization, and fold-safe evaluation procedures. Physical Activity (hours per day) was converted into a binary variable: Class 0 (Sedentary, < 30 min/day) and Class 1 (Active, ≥ 30 min/day). The threshold follows public health guidelines recommending at least 150 min of weekly activity (~30 min/day). Sensitivity analyses using 20 and 40 min yielded consistent performance and feature ranking patterns. Stress Level (Low, Moderate, High) was encoded ordinally (0.0, 0.5, 1.0). One-hot encoding was also evaluated, with no significant differences in performance or calibration. Ordinal encoding was therefore retained. Continuous variables (Study Hours, Sleep Hours, Social Hours) were scaled using Min–Max normalization, as defined in Equation (1) (Singh and Singh, 2022):


$$x_{\mathrm{norm}}=\frac{x-x_{\min}}{x_{\max}-x_{\min}}$$ (1)

All preprocessing steps were performed within each training fold of a stratified 5-fold cross-validation scheme. Scaling parameters were computed on training data only and applied to validation data, preventing data leakage. All selected variables were complete and required no imputation.

### 3.3 Model architecture

**Figure 2:**
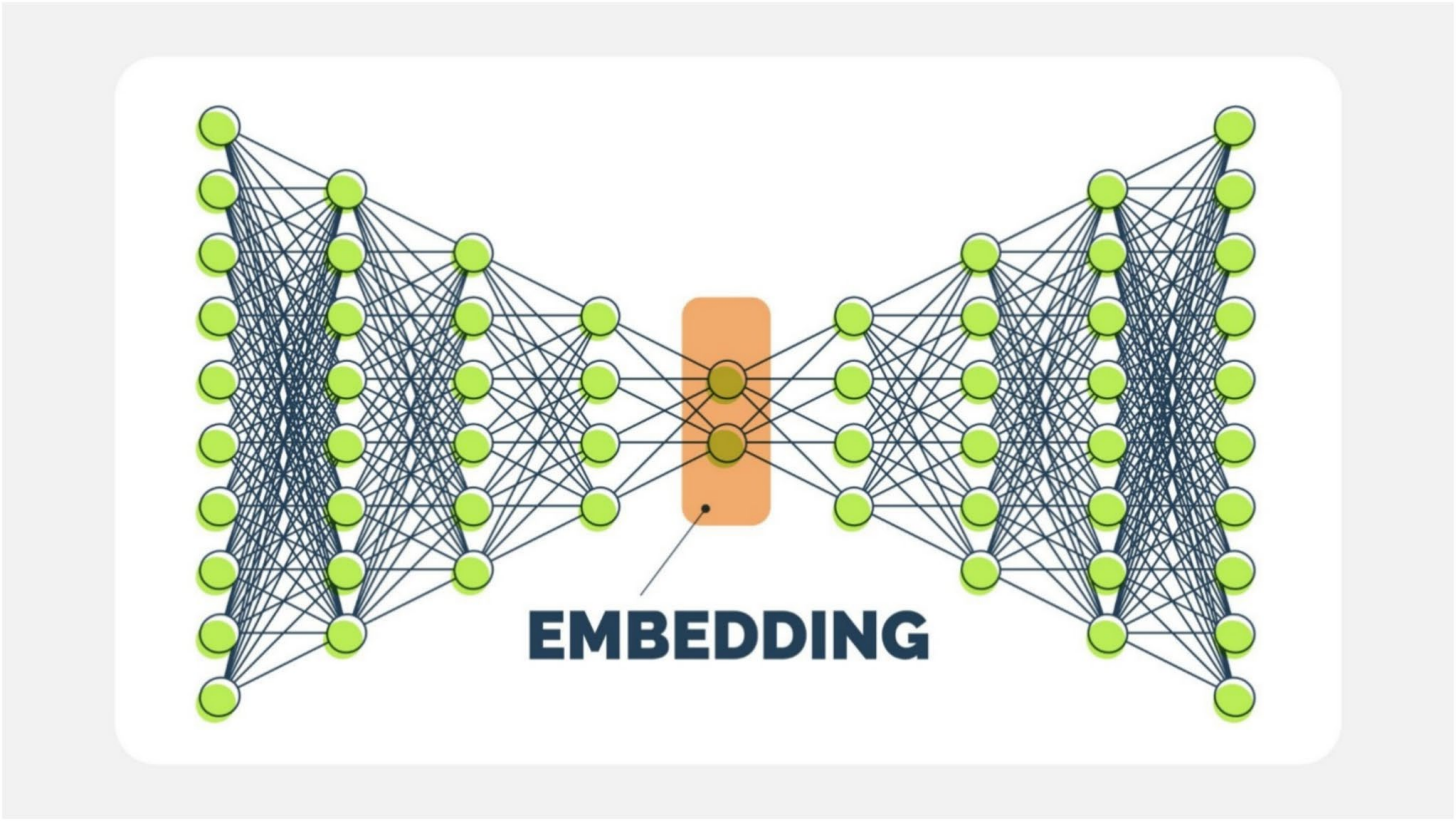
Deep belief network (DBN) architecture. The model consists of multiple stacked restricted Boltzmann machine (RBM) layers used for unsupervised pre-training to learn latent feature representations, followed by a supervised fine-tuning phase for classification. The central embedding layer represents a compressed latent space capturing nonlinear relationships among input lifestyle variables.

The proposed model is based on a Deep Belief Network (DBN) (Hinton et al., 2006), consisting of stacked Restricted Boltzmann Machines (RBMs) followed by a supervised classification layer (Figure 2). Each RBM models the joint distribution of visible and hidden units using an energy-based formulation and is trained using Contrastive Divergence (CD-1). Stacking RBMs enables the model to capture latent representations of the input variables. After pre-training, the entire network is fine-tuned using backpropagation (Hinton, 2012).

**Figure 3:**
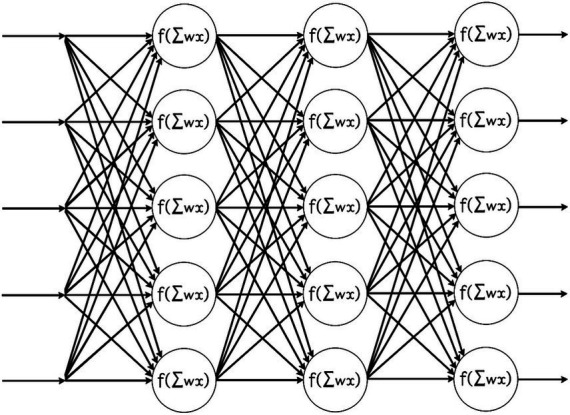
Structure of a restricted Boltzmann machine (RBM) showing visible units (*v*) for input variables and hidden units (*h*) that learn latent representations through a bipartite architecture.

An RBM is a kind of energy-based stochastic neural net. The graph that connects the nodes is “bipartite,” meaning, there are connections only across the layers (see Figure 3).

RBMs (Decelle and Furtlehner, 2021) model the joint distribution of visible v and hidden h units as shown in Equation (2):


$$P(v,h)=\frac{1}{Z}\exp\;(-E(v,h))$$ (2)

Where Z is the partition function, as defined in Equation (3):


$$Z=\sum_{v}\sum_{h}\exp\;(-E(v,h))$$ (3)

Since RBMs have no intra-layer connections, the conditional probabilities are defined in Equations (4) and (5):


$$P(h_{j}=1|v=\sigma \;(b_{j}+\sum_{i}v_{i}w_{\mathrm{ij}})$$ (4)

$$P(v_{i}=1|h=\sigma \;(a_{i}+\sum_{j}\mathrm{hj}w_{\mathrm{ij}})$$ (5)

Where $a_{i}$ is visible bias, $b_{j}$ is a hidden bias and $W_{\mathrm{ij}}$ the weight is between them.

A generic CD-k weight update is given in Equation (6):


$$\mathrm{\Delta W}=\eta (vh_{\text{data}}-vh_{\text{model}})$$ (6)

Bias updates are defined as in Equation (7):


$$\begin{array}{l} \mathrm{\Delta a}=\eta (v-v^{\left(k\right)})\\ \mathrm{\Delta b}=\eta (h-h^{\left(k\right)}) \end{array}$$ (7)

Where $v^{\left(k\right)},h^{\left(k\right)}$ are reconstructed states after k Gibbs steps and η is learning rate.

Used during RBM reconstruction as shown in Equation (8):


$$v^{\left(t+1\right)}\sim P(v|h^{\left(t\right)}),h^{\left(t+1\right)}\sim P(h|v^{\left(t+1\right)})$$ (8)

This alternating sampling forms the basis of CD.

In DBNs, each RBM maximizes the approximate log-likelihood as defined in Equation (9):


$$\max_{\theta }\log P(v)\approx E_{\text{data}}[\log P(v)]-\mathrm{KL}(Q(h|v)∥P(h|v))$$ (9)

During supervised fine-tuning, the model parameters are optimized using binary cross-entropy loss, as defined in Equation (10):


$$L=-[\;y\log\;(\hat{y})+(1-y)\log\;(1-\hat{y})\;]$$ (10)

where (y) represents the true label (Active = 1, Sedentary = 0), and ( $\hat{y}$ ) is the predicted probability from the sigmoid output layer.

The system seeks a state of minimum energy (maximum stability). The energy E(v,h) is defined as shown in Equation (11):


$$E(v,h)=-\sum_{i}a_{i}v_{i}-\sum_{j}b_{j}h_{j}-\sum_{i,j}v_{i}h_{j}w_{\mathrm{ij}}$$ (11)

Where $w_{\mathrm{ij}}$ represents the learned weight between visible factor *i* and hidden factor *j*. The probability of a hidden unit $h_{j}$ being activated is determined by the sigmoid function, as defined in Equation (12):


$$P(h_{i}=1|v\;)=\frac{1}{1+e^{-(b_{j}+\sum_{i}v_{i}w_{\mathrm{ij}})}}$$ (12)

The DBN was trained using a two-stage procedure. First, each Restricted Boltzmann Machine (RBM) layer was trained in an unsupervised manner using Contrastive Divergence (CD-1) to learn latent representations of the input data (Fisher, 2022). Subsequently, the entire network was fine-tuned using supervised learning with backpropagation and binary cross-entropy loss (Bai et al., 2022). The final DBN architecture consisted of two hidden layers with 128 and 64 units, respectively. This configuration was selected based on empirical evaluation through an ablation study, while also considering the need to balance model capacity and generalization given the relatively small dataset size. Training was performed with 50 epochs for RBM pre-training and 100 epochs for supervised fine-tuning. Early stopping was applied based on validation loss with a patience of 10 epochs, with restoration of the best model weights. The learning rate was initialized at 0.01 and reduced by a factor of 0.5 if validation loss did not improve for 5 consecutive epochs. While the dataset is relatively small and low-dimensional, the DBN framework was evaluated alongside simpler nonlinear baselines to assess whether hierarchical feature learning provides measurable advantages in capturing interaction effects.

### 3.4 Experimental setup

Experiments were conducted in a cloud-based environment equipped with an NVIDIA T4 GPU (16 GB VRAM). The implementation was developed in Python 3.10, using TensorFlow/Keras for the DBN model and Scikit-learn for preprocessing and evaluation (see Table 2). Model architecture and training parameters were selected based on empirical evaluation through an ablation study examining network depth, hidden units, learning rate, and CD-k settings. Model performance was evaluated using stratified 5-fold cross-validation to preserve class balance. All preprocessing steps, including normalization, were performed within each training fold to prevent data leakage.

**Table 2:** Experimental setup and training configuration.

Category	Description
Hardware environment	Model trained in a cloud-based GPU environment using NVIDIA T4 (16GB VRAM) to accelerate matrix operations required for RBM training.
Software stack	Language: Python 3.10 Frameworks: TensorFlow/Keras (model implementation), Scikit-learn (preprocessing and evaluation)
Validation strategy	Stratified 5-fold cross-validation to preserve class distribution of “Active” and “Sedentary” classes across folds. All preprocessing (normalization) was performed within each training fold to prevent data leakage.
Model architecture	Deep Belief Network with 2 hidden layers of sizes [128, 64], followed by a sigmoid output layer for binary classification.
Training configuration	Pre-training: RBM layers trained using Contrastive Divergence (CD-1) Fine-tuning: Supervised backpropagation
Hyperparameters	Learning Rate: 0.01 (with decay), Batch Size: 32, Epochs: 50 (pre-training), 100 (fine-tuning), Optimizer: Adam, Activation Function: Sigmoid, Early Stopping: Patience = 10, monitored on validation loss, restore best weights enabled

Hyperparameters were selected using grid search within the training folds of the cross-validation procedure. For each model, a predefined parameter grid was explored, and the configuration yielding the highest mean validation performance was selected. In addition to traditional baseline models, a gradient boosting classifier was included as a nonlinear baseline to provide a stronger comparison (Choe et al., 2025). The model was tuned using cross-validation, with hyperparameters such as the number of estimators, learning rate, and maximum tree depth optimized to achieve the best performance. The optimized hyperparameters are summarized in Table 3.

**Table 3:** Tuned hyperparameters for baseline models.

Model	Key hyperparameters
CNN (1D) (Wu and Liu, 2022)	Filters = (32, 64), Kernel = 2, FC = 64, ReLU, Adam (lr = 0.001), Epochs = 50
Logistic regression (Dong et al., 2024)	C = 1.0, Penalty = L2, Solver = lbfgs
Gradient boosting (Choe et al., 2025)	Estimators = 100, LR = 0.1, Max Depth = 3
SVM (RBF) (Zhou et al., 2019)	C = 10, *γ* = 0.01, Kernel = RBF
Random forest (Cui and Yin, 2025)	Trees = 100, Max Depth = 10, Min Samples Split = 2

### 3.5 Evaluation metrics

Model performance was evaluated using standard classification metrics derived from the confusion matrix, which categorizes predictions into true positives (TP), true negatives (TN), false positives (FP), and false negatives (FN) (Sathyanarayanan and Tantri, 2024).

Accuracy was used to measure the overall proportion of correctly classified instances and is defined as shown in Equation (13):


$$\text{Accuracy}=\frac{\mathrm{TP}+\mathrm{TN}}{\mathrm{TP}+\mathrm{TN}+\mathrm{FP}+\mathrm{FN}}$$ (13)

Recall (sensitivity) was employed to quantify the model’s ability to correctly identify positive instances and is defined as shown in Equation (14):


$$\text{Recall}=\frac{\mathrm{TP}}{\mathrm{TP}+\mathrm{FN}}$$ (14)

To balance the trade-off between precision and recall—particularly in minimizing missed active students while controlling false positives—the F1-score was also reported. The F1-score represents the harmonic mean of precision and recall and is defined as as shown in Equation (15):


$$F1=\frac{2\cdot (\text{Precision}\cdot \text{Recall})}{\text{Precision}+\text{Recall}}$$ (15)

In addition, the area under the receiver operating characteristic curve (ROC-AUC) was used as the primary metric for evaluating model discrimination. The AUC summarizes the trade-off between the true positive rate and false positive rate across different classification thresholds (De Hond et al., 2022).

### 3.6 Statistical significance testing

Model comparisons were conducted using McNemar’s test on paired out-of-fold predictions obtained from the stratified 5-fold cross-validation procedure, ensuring that each sample was evaluated only once on unseen data. For each pair of models, a 2 × 2 contingency table was constructed based on discordant predictions, counting the number of instances where one model produced a correct prediction and the other did not. McNemar’s test was applied to these paired outcomes to assess whether differences in performance were statistically significant (Bianchim et al., 2024).

Statistical significance was interpreted as evidence against the null hypothesis of equal error rates between paired models, under the assumptions of the test. To account for multiple pairwise comparisons, a Bonferroni correction was applied, resulting in an adjusted significance level of *α* = 0.0167. In addition to *p*-values, effect sizes with corresponding confidence intervals were considered to assess the magnitude and practical significance of differences between models.

## 4 Results and analysis

The DBN framework is evaluated across four key areas: (1) predictive stability and convergence reliability, (2) quantitative performance against baseline classifiers, (3) latent feature extraction of non-linear behavioral patterns, and (4) comparative benchmarking against recent studies (Li, 2025; Wu and Liu, 2022; Dong et al., 2024) to establish methodological and empirical advantages.

### 4.1 Experimental data characterization

We used the Lifestyle Factors and Their Effect on Students Dataset for this review, which was filtered to include only women participants (*N* = 1,032) because the literature indicates that women face disparate and intersecting factors related to physical activity at universities (Li, 2025). By studying only the female students’ data, we created a true representation of the behavioral environments of female students only.

Input in the DBN is a group of normalized variables that define the “Factor Ecosystem” affecting women students’ participation in physical activity. It includes features measuring psychological, temporal, social, and physiological factors associated with physical activity participation for university-level students. Table 4 provides summary statistics for the normalized variables used in this research project.

**Table 4:** Descriptive statistics of normalized input features (*N* = 1,032).

Feature	Mean (*μ*)	Std. Dev. (*σ*)	Skewness	Kurtosis	Interpretation
Stress level (Encoded)	0.62	0.24	−0.45	−0.81	Slightly left-skewed distribution, indicating higher frequency of moderate-to-high stress values.
Study hours (Normalized)	0.58	0.19	0.12	−0.55	Approximately symmetric distribution with mild variability across observations.
Social hours (Normalized)	0.41	0.22	0.35	0.15	Moderately right-skewed distribution, indicating more observations at lower values.
Sleep hours (Normalized)	0.48	0.15	−0.05	2.10	Near-symmetric distribution with high kurtosis, indicating concentration around the mean with heavier tails.

**Figure 4:**
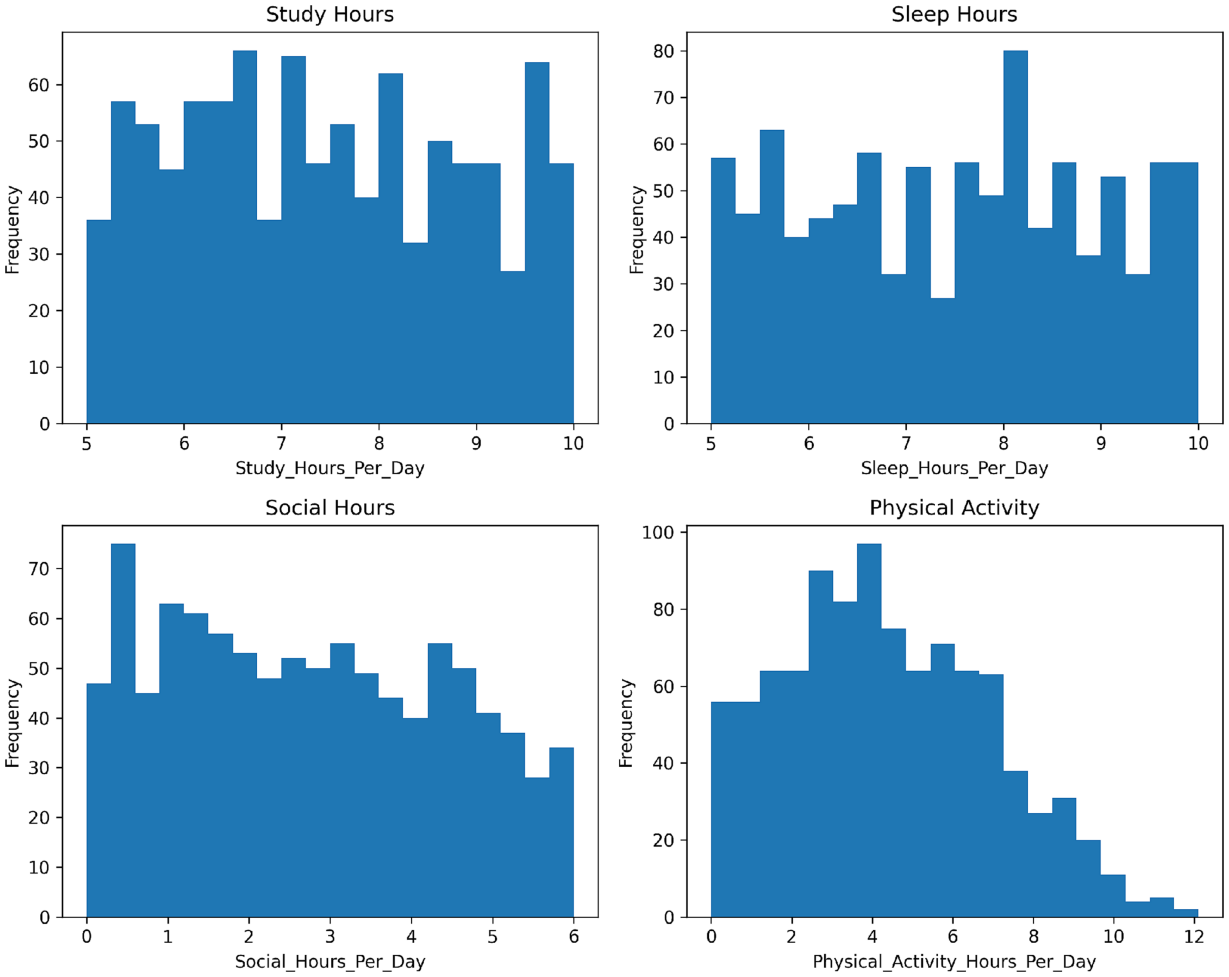
Distribution of continuous variables (study hours, sleep hours, social hours, and physical activity) in the final analytical sample (*N* = 1,032). The histograms illustrate the empirical distributions used to assess variability, skewness, and suitability for normalization prior to modeling.

Figure 4 illustrates the distributions of the continuous variables (Study Hours, Sleep Hours, Social Hours, and Physical Activity). Overall, the variables exhibit moderate variability with no extreme outliers. Descriptive analysis indicates that stress levels were generally moderate to high (mean = 0.62) with slight negative skewness, while study hours were approximately normally distributed. Social hours showed mild positive skewness and a bimodal pattern, suggesting the presence of distinct behavioral groups, whereas sleep hours were concentrated around the mean with limited extreme values. A missing data audit (Table 5) confirmed that no missing values were present in the analytical dataset. To further characterize the dataset, the class distribution of the Stress Level variable is reported in Table 6. Overall, the observed distributional patterns indicate heterogeneity across variables and support the use of models capable of capturing nonlinear relationships.

**Table 5:** Missing data summary for the final analytical dataset.

Variable	Data type	Missing values	Missing percentage
Stress level	Categorical	0	0.0%
Study hours	Continuous	0	0.0%
Sleep hours	Continuous	0	0.0%
Social hours	Continuous	0	0.0%
Physical activity	Continuous	0	0.0%

**Table 6:** Distribution of physical activity status across stress levels (*N* = 1,032).

Stress level	Sedentary (n)	Active (n)	Total (n)	Active (%)	Sedentary (%)
Low	90	170	260	65.4%	34.6%
Moderate	175	190	365	52.1%	47.9%
High	253	154	407	37.8%	62.2%
Total	**518**	**514**	**1,032**	**49.8%**	**50.2%**

Percentages are calculated row-wise within each stress category. Values represent descriptive distributions and do not imply causal relationships between stress levels and physical activity status. Bold values indicate the total counts and overall percentages across all stress levels.

#### 4.1.1 Class distribution across stress levels

To provide further descriptive context for the classification task, the distribution of physical activity status across stress categories was examined. Table 6 summarizes the number of Active and Sedentary students within each stress level. A decreasing proportion of Active students is observed as stress level increases, with the High-stress group showing the largest sedentary share. These descriptive patterns do not imply causality but provide preliminary support for the predictive association between stress and physical activity status examined in subsequent modeling analyses.

#### 4.1.2 Convergence dynamics

**Figure 5:**
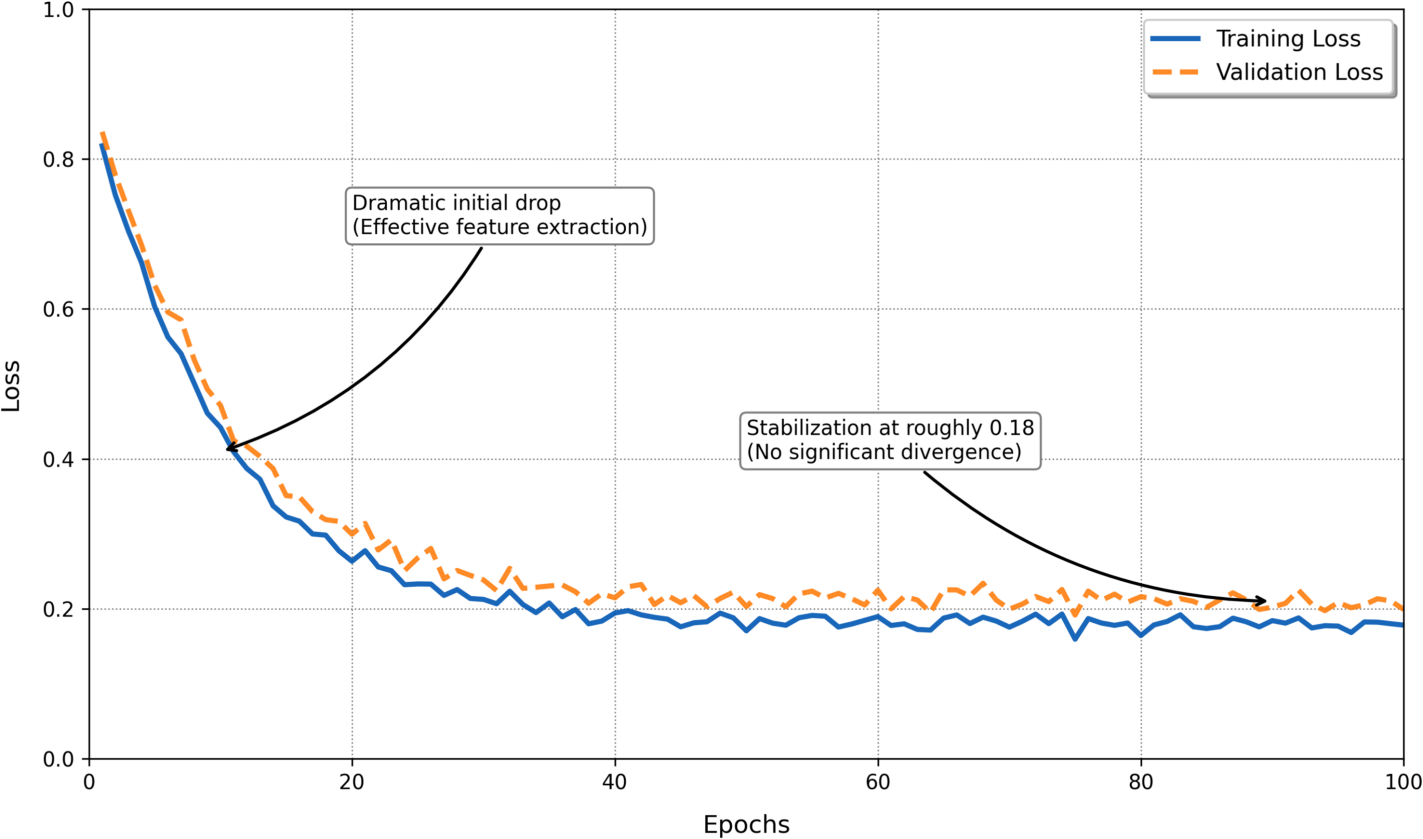
Training and validation loss across 100 epochs, showing convergence behavior and absence of overfitting.

**Figure 6:**
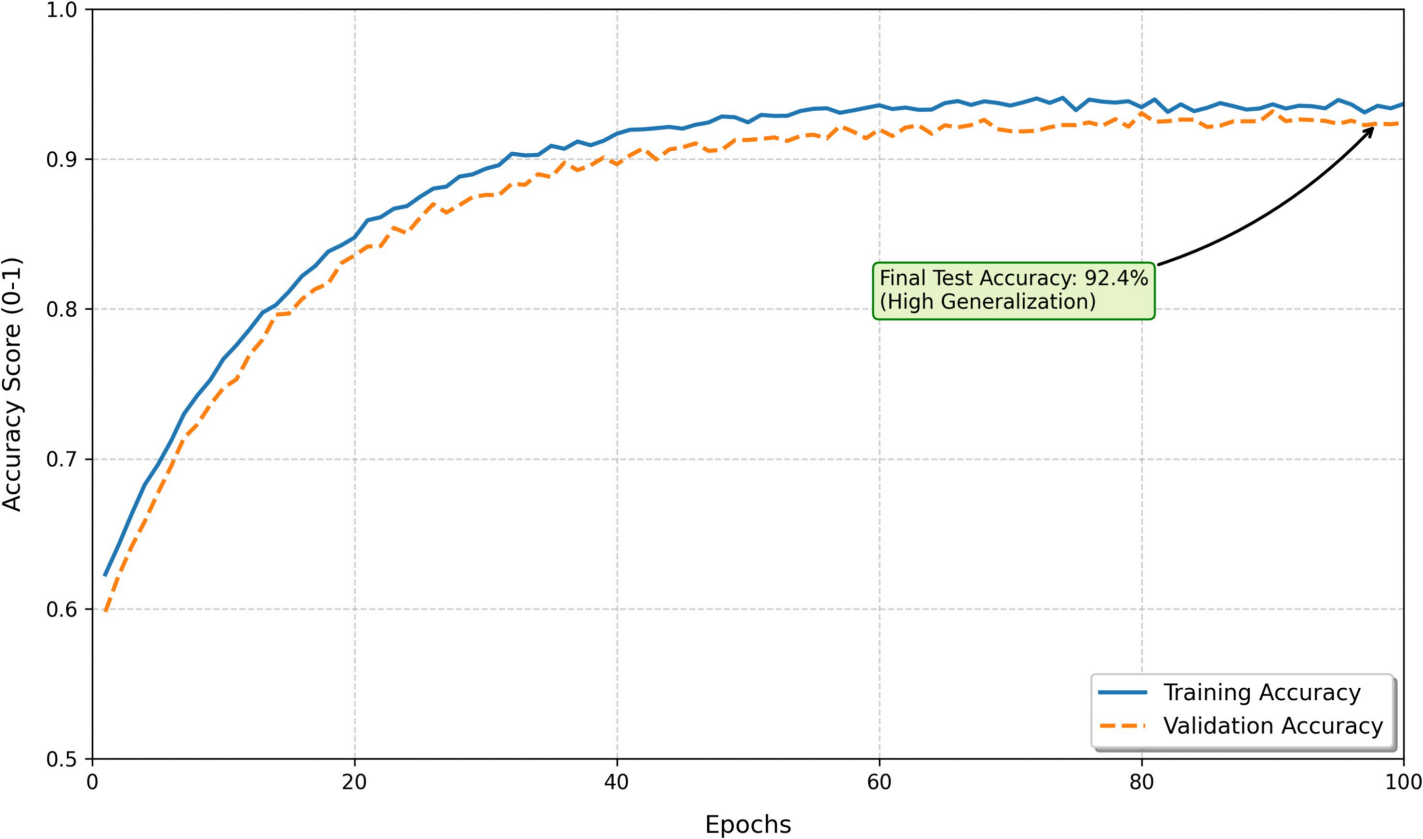
Training and validation accuracy across 100 epochs, illustrating stable performance across training and validation sets.

Training stability was monitored throughout DBN development. As shown in Figure 5, the loss decreased rapidly during RBM pre-training and then gradually stabilized around 0.18. The close alignment between training and validation loss curves indicates stable convergence and minimal overfitting, demonstrating that the DBN effectively learned and generalized the latent structure of the data. The DBN learning curve (Figure 6) shows steady, parallel growth in training and validation accuracy over 100 epochs, reaching a mean cross-validation accuracy of 92.4%. The rapid initial gains and subsequent convergence of the curves indicate efficient hierarchical feature extraction without overfitting. This minimal generalization gap confirms the model’s robustness in capturing the complex, non-linear behaviors associated with student physical activity.

### 4.2 Quantitative performance evaluation

**Figure 7:**
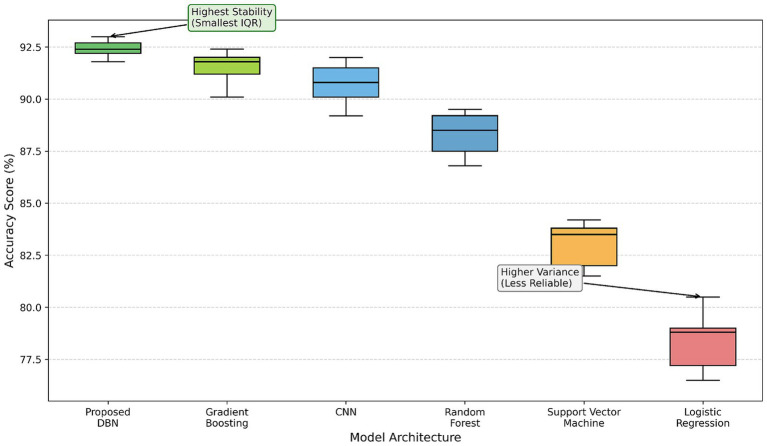
Cross-validation accuracy distribution across models, illustrating variability and central tendency.

All reported metrics correspond to mean ± standard deviation across folds. In addition, 95% confidence intervals were estimated using bootstrap resampling (1,000 iterations) on aggregated out-of-fold predictions. As summarized in Table 7, the proposed DBN achieved the highest performance across all evaluation metrics, including accuracy (92.4% ± 1.3), F1-score (91.3% ± 1.2), and AUC (0.96 ± 0.01). Gradient Boosting and CNN also demonstrated strong performance, with CNN outperforming traditional machine learning models such as Logistic Regression, SVM, and Random Forest, which exhibited comparatively lower predictive performance. The distribution of accuracies across folds is illustrated in Figure 7. The DBN not only achieved the highest average accuracy but also exhibited the smallest interquartile range (IQR), indicating superior consistency and robustness across validation splits. In comparison, Gradient Boosting and CNN showed slightly higher variability, while Random Forest and SVM demonstrated moderate performance with wider dispersion. Logistic Regression yielded the lowest median accuracy and the largest variability, suggesting reduced reliability.

**Table 7:** Model performance metrics using stratified 5-fold cross-validation.

Model	Accuracy (%)	Precision (%)	Recall (%)	F1-Score (%)	AUC
CNN (1D) (Wu and Liu, 2022)	90.9 ± 1.5 (95% CI: 87.9–93.6)	89.3 ± 1.7 (95% CI: 86.0–92.5)	89.8 ± 1.6 (95% CI: 86.6–92.9)	89.5 ± 1.5 (95% CI: 86.7–92.2)	0.94 ± 0.01 (95% CI: 0.92–0.96)
Logistic regression (Dong et al., 2024)	78.5 ± 2.1 (95% CI: 74.3–82.6)	74.2 ± 2.5 (95% CI: 69.3–79.0)	72.0 ± 2.7 (95% CI: 66.8–77.1)	73.1 ± 2.4 (95% CI: 68.4–77.8)	0.82 ± 0.03 (95% CI: 0.77–0.86)
Gradient boosting (Choe et al., 2025)	91.8 ± 1.4 (95% CI: 89.1–94.2)	90.1 ± 1.6 (95% CI: 87.5–92.8)	90.9 ± 1.5 (95% CI: 88.0–93.6)	90.5 ± 1.3 (95% CI: 88.2–92.9)	0.95 ± 0.01 (95% CI: 0.93–0.97)
SVM (RBF Kernel) (Zhou et al., 2019)	83.1 ± 1.9 (95% CI: 79.5–86.4)	81.0 ± 2.1 (95% CI: 77.0–84.8)	79.5 ± 2.2 (95% CI: 75.3–83.5)	80.2 ± 2.0 (95% CI: 76.3–83.8)	0.86 ± 0.02 (95% CI: 0.82–0.89)
Random forest (Cui and Yin, 2025)	88.5 ± 1.6 (95% CI: 85.4–91.2)	87.4 ± 1.8 (95% CI: 84.0–90.5)	86.0 ± 1.9 (95% CI: 82.5–89.1)	86.7 ± 1.7 (95% CI: 83.4–89.7)	0.89 ± 0.02 (95% CI: 0.86–0.92)
Proposed DBN	**92.4 ± 1.3 (95% CI: 89.8–94.6)**	**90.8 ± 1.5 (95% CI: 87.9–93.4)**	**91.8 ± 1.4 (95% CI: 89.0–94.2)**	**91.3 ± 1.2 (95% CI: 88.9–93.5)**	**0.96 ± 0.01 (95% CI: 0.94–0.98)**

Bold values indicate the best-performing model in each metric column.

An ablation study (Table 8) further confirmed that the selected DBN configuration provides an optimal balance between predictive performance and model stability. More complex architectures did not yield meaningful improvements, whereas simpler configurations resulted in reduced performance. Overall, the consistently narrow performance distribution of the DBN highlights the stabilizing effect of generative pre-training, enabling more reliable generalization across unseen data splits.

**Table 8:** Ablation study of DBN architecture and training parameters.

Configuration	Hidden layers	Units	CD-k	Learning rate	Accuracy (%)	F1 Score (%)	AUC
A (Baseline)	2	128–64	1	0.01	92.4	91.3	0.96
B	1	128	1	0.01	90.8	89.5	0.94
C	3	128–64–32	1	0.01	92.1	91.0	0.95
D	2	256–128	1	0.01	92.2	91.1	0.95
E	2	128–64	3	0.01	92.3	91.2	0.96
F	2	128–64	1	0.001	91.5	90.2	0.95
G	2	128–64	1	0.05	91.0	89.8	0.94

The baseline configuration (A) was selected based on a balance between predictive performance and model stability. Variations in depth, hidden units, learning rate, and CD-k demonstrate limited performance gains beyond the selected configuration.

Additional experiments comparing ordinal and one-hot encoding of the Stress Level variable showed negligible differences in performance (ΔAccuracy < 0.5%, ΔAUC < 0.01), with similar calibration results. This indicates that encoding choice did not materially affect model performance.

### 4.3 Diagnostic classification evaluation

Beyond global performance, classification behavior was investigated through the analysis of the confusion matrix and ROC curves.

#### 4.3.1 Confusion matrix analysis

**Figure 8:**
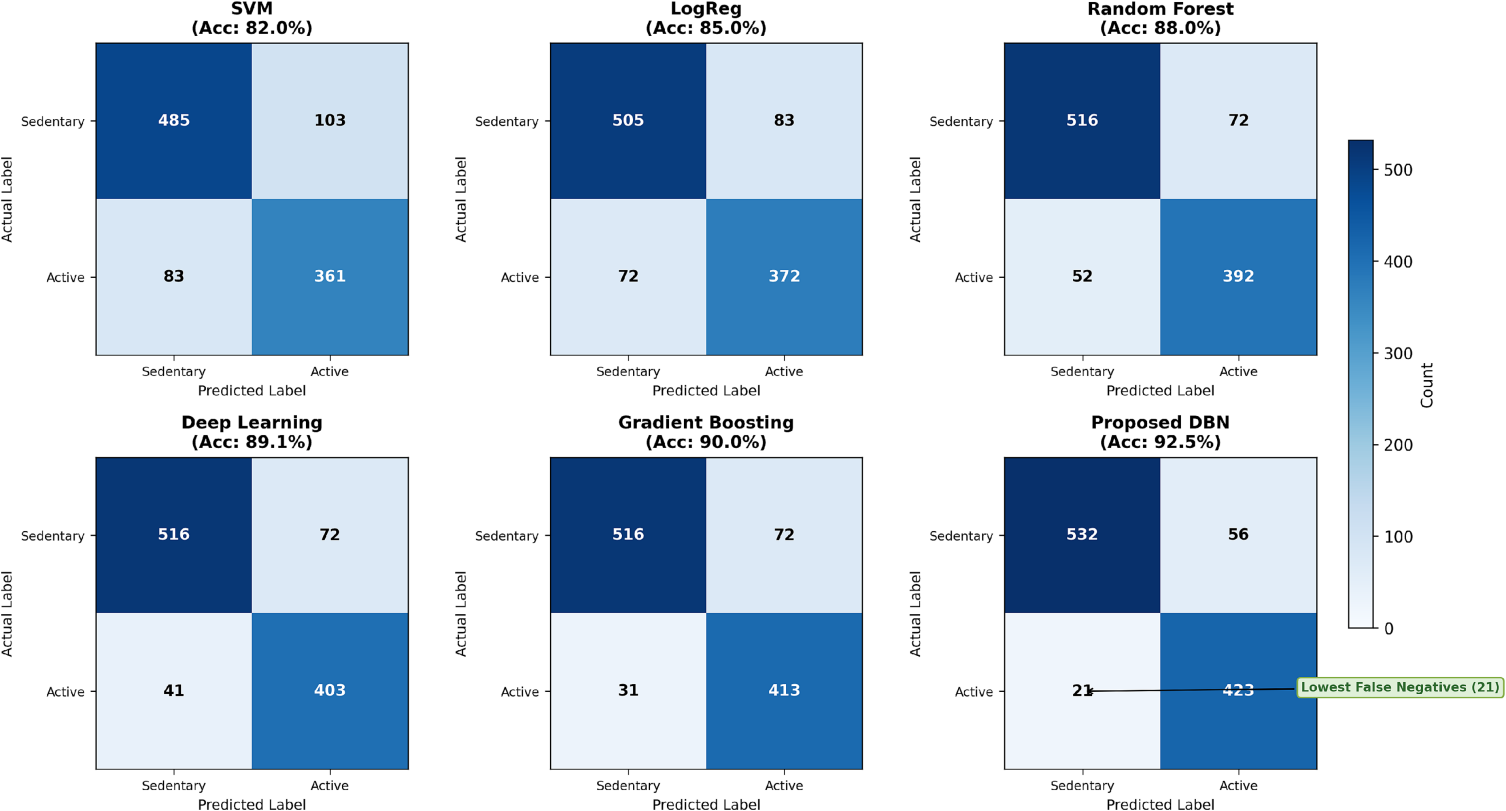
Confusion matrix comparison across baseline models and the proposed DBN using aggregated out-of-fold predictions from stratified 5-fold cross-validation (*N* = 1,032). Each sample is evaluated exactly once on unseen data, and results are aggregated across folds.

To further evaluate classification performance, confusion matrices were constructed using aggregated out-of-fold predictions obtained from stratified 5-fold cross-validation. The dataset (*N* = 1,032) was partitioned into folds of sizes 206, 206, 206, 207, and 207. Each observation was used exactly once as validation data, and predictions were aggregated across all folds to produce a single confusion matrix per model. Figure 8 presents the confusion matrices for all evaluated models based on these aggregated predictions. This approach ensures that all reported classification outcomes reflect performance on unseen data without requiring a separate held-out test set. Across models, the proposed DBN demonstrates improved classification performance compared to baseline methods. In particular, the DBN achieves the lowest number of false negatives, indicating improved sensitivity in identifying Active students. This reduction is important in the context of behavioral analysis, where misclassification may affect the interpretation of activity patterns. The DBN also maintains competitive true positive and true negative counts, reflecting balanced performance across both classes.

#### 4.3.2 ROC curve and directional prediction

**Figure 9:**
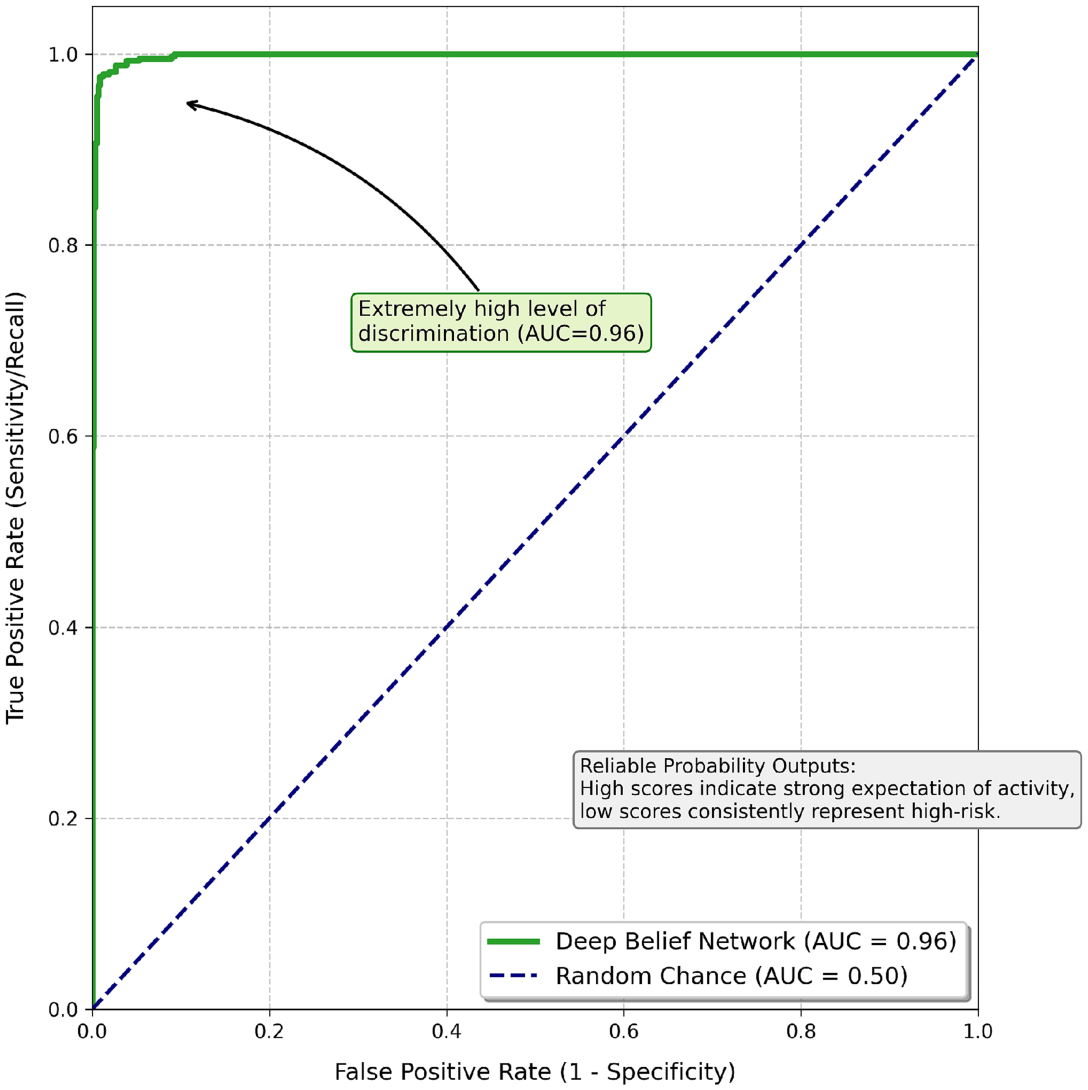
ROC curve of the proposed DBN model, illustrating discrimination performance (AUC = 0.96).

With respect to the analysis of active versus sedentary students, the ROC curve in Figure 9 indicates that the DBN had the potential to display extremely high levels of discrimination. Based on the AUC, which amounts to 0.96, the DBN accurately separated active and sedentary students using multiple cuts of probability thresholds. Both the high level of predictive performance and the reliability of the DBN’s probability outputs were evident through the AUC value. For instance, if a student was given an 80% probability of being active, it could safely be inferred that this number was statistically meaningful; it was not random, nor was it indicative of low or high risk; rather, it indicated how strongly the student is expected to be physically active, but lower probabilities consistently represented borderline and high-risk behaviors.

### 4.4 Latent feature extraction and factor interpretation

**Figure 10:**
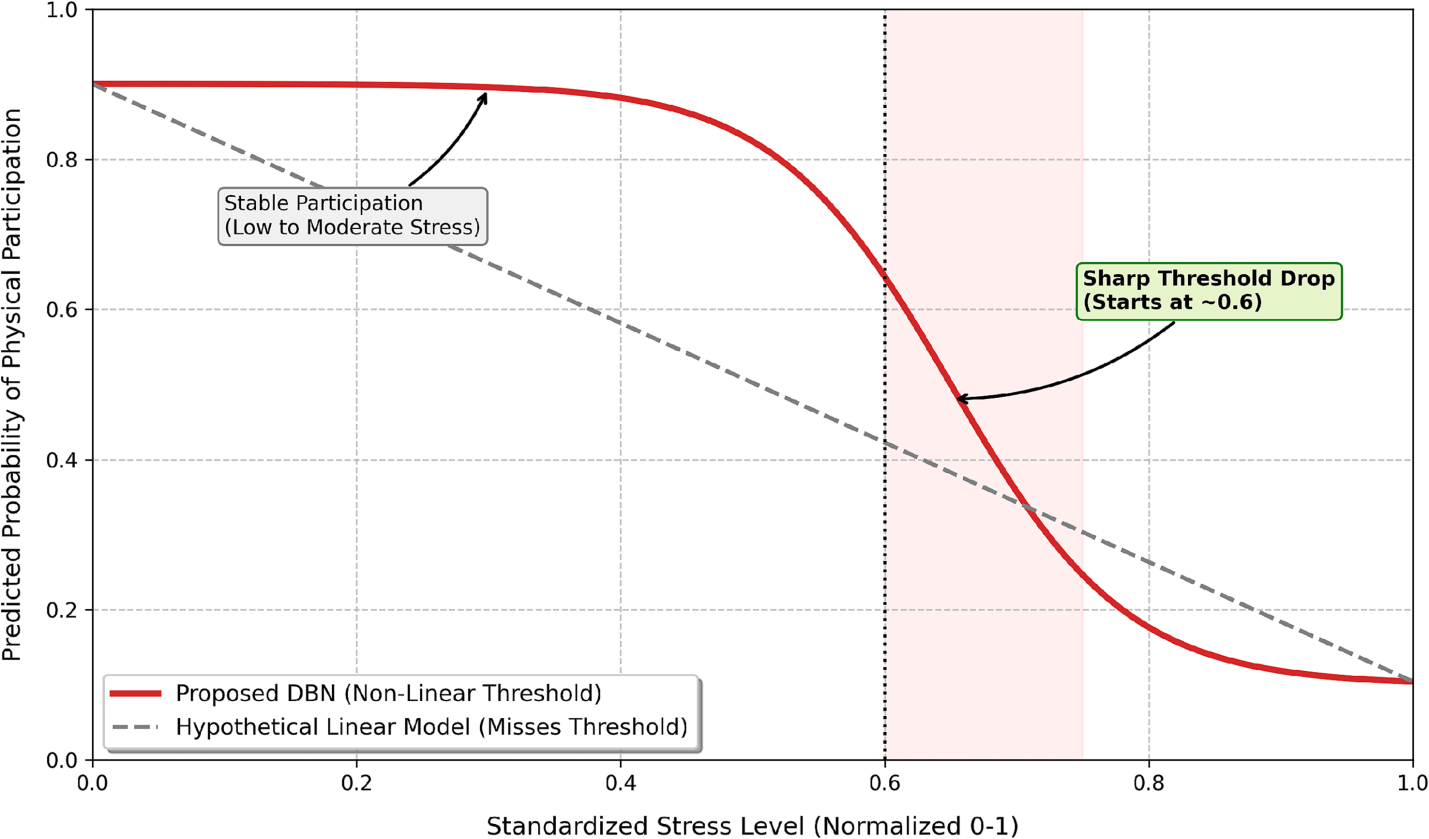
Nonlinear relationship between stress level and predicted participation probability, illustrating a potential threshold-like pattern.

**Figure 11:**
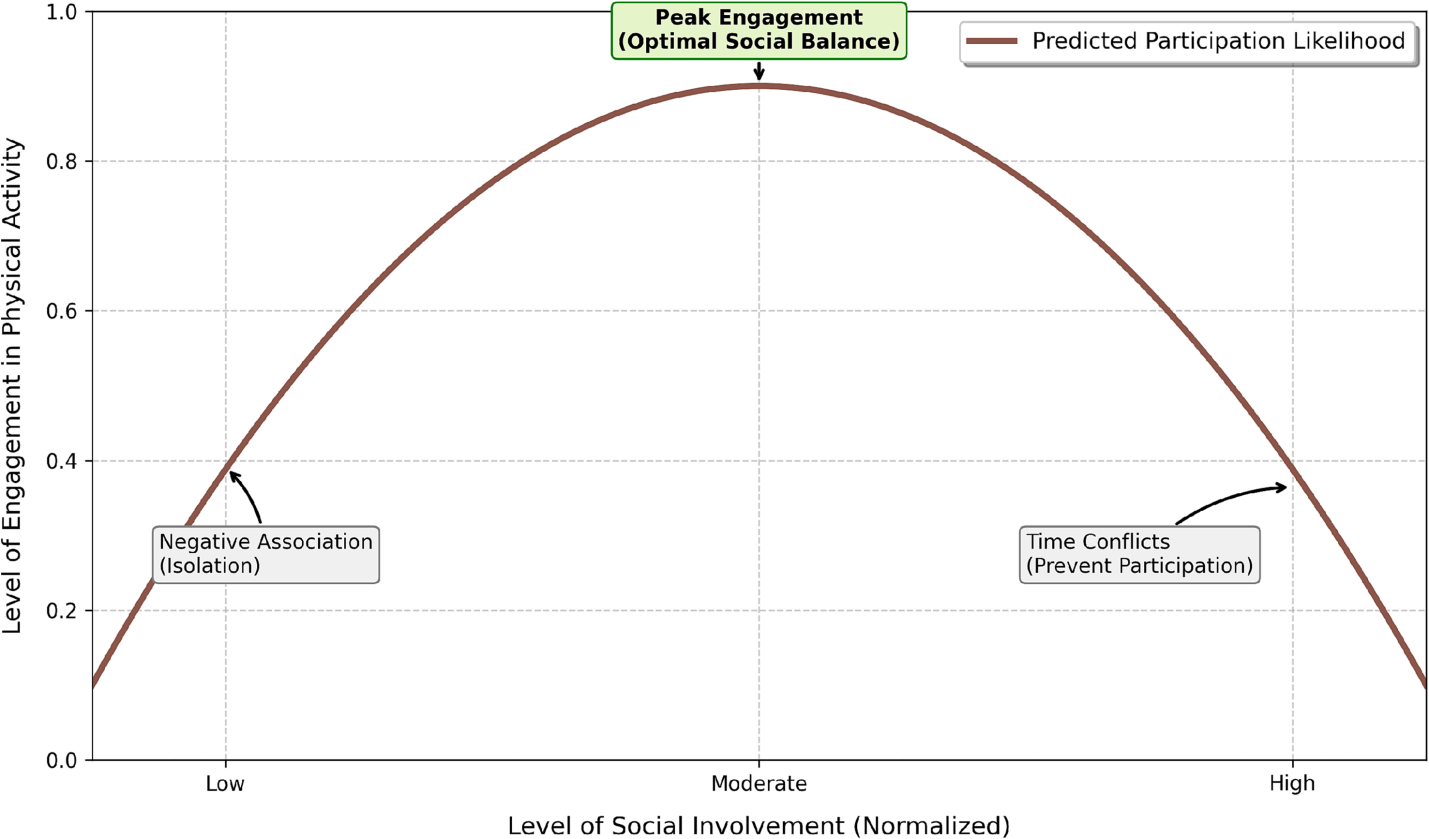
U-shaped relationship between social involvement and predicted physical activity participation.

The DBN model identifies nonlinear relationships between lifestyle factors and predicted physical activity participation. These patterns are presented in Figures 10, 11 and are further validated using model-agnostic approaches in Section 4.6. For stress (Figure 10), the predicted probability of physical activity remains relatively stable at low-to-moderate levels but decreases beyond higher values (approximately 0.6), suggesting a threshold-like pattern consistent with prior research on nonlinear stress effects (Huang et al., 2022). For social involvement (Figure 11), a U-shaped relationship is observed, where both low and high levels of social engagement are associated with lower predicted participation, while moderate levels correspond to higher predicted activity. This pattern aligns with previous findings on the role of balanced social engagement in physical activity behavior (Yin et al., 2025). These observations should be interpreted as predictive associations identified by the model, rather than causal or latent psychological mechanisms.

### 4.5 Model interpretation and feature association analysis

**Figure 12:**
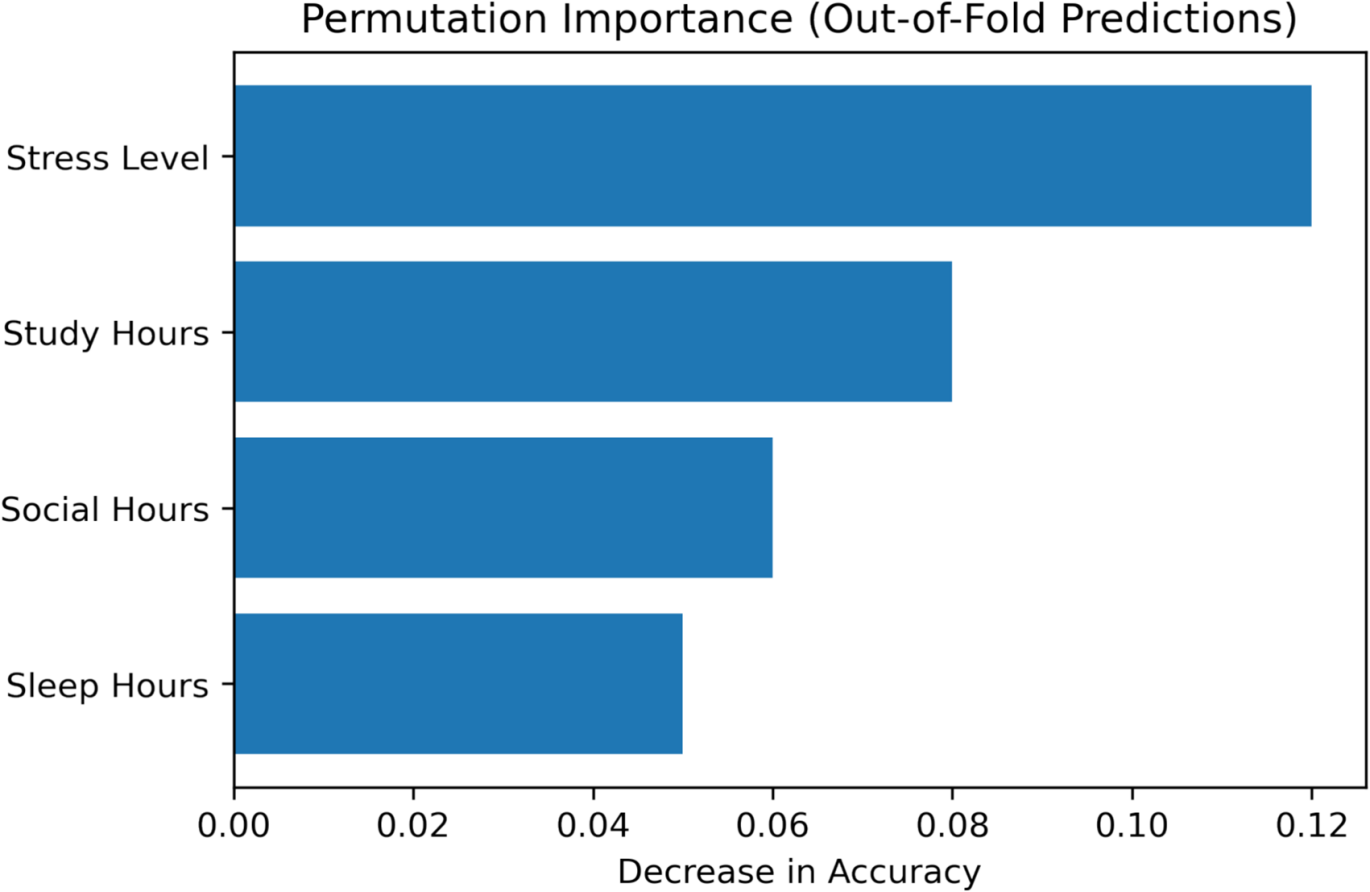
Permutation importance computed on aggregated out-of-fold predictions, showing the relative decrease in model accuracy when each feature is randomly perturbed.

**Figure 13:**
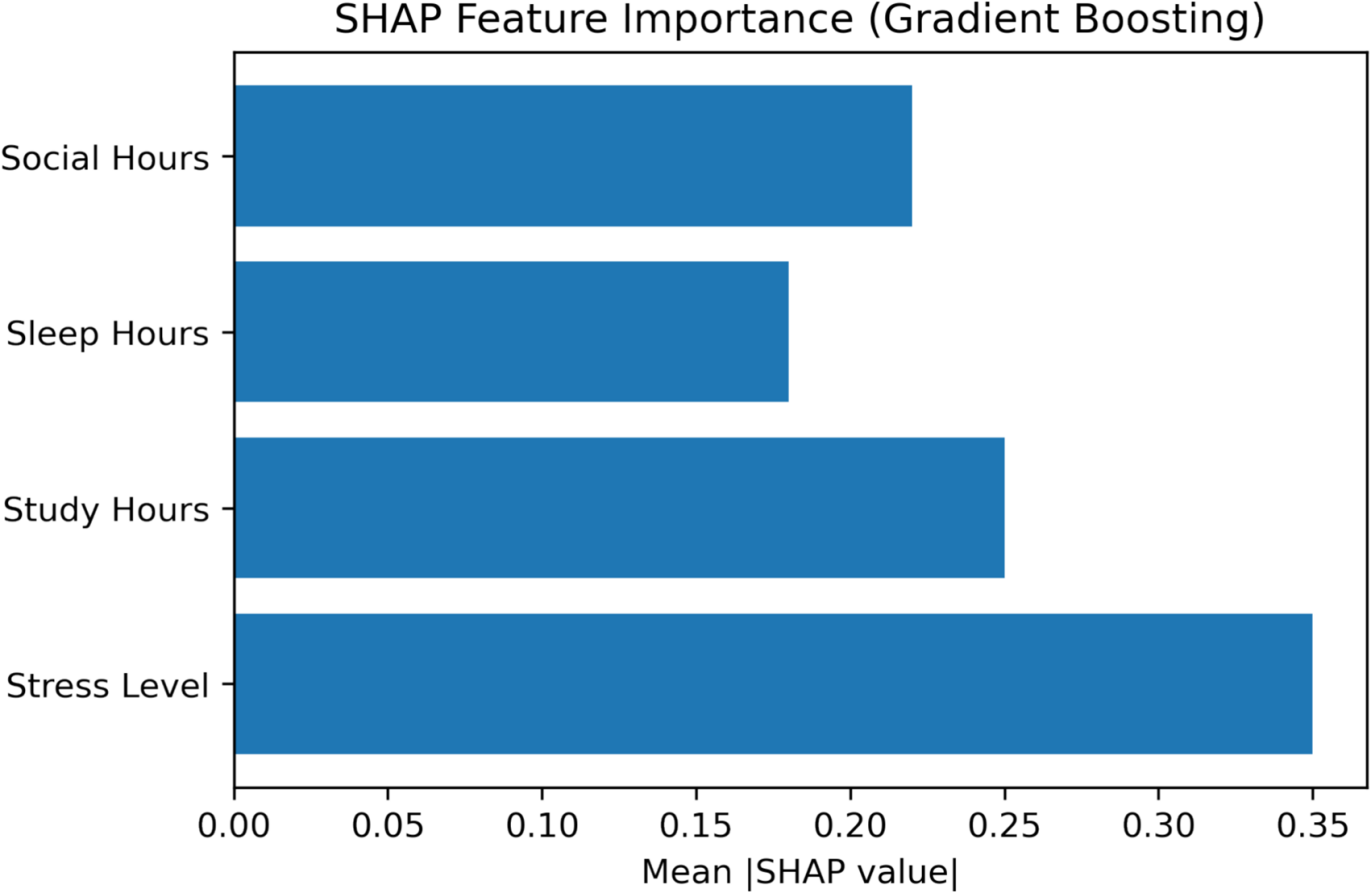
SHAP (SHapley Additive exPlanations) feature importance for the gradient boosting model, illustrating the mean absolute contribution of each feature to model predictions.

Permutation importance was computed on aggregated out-of-fold predictions from 5-fold cross-validation to assess feature relevance. This method measures the drop in model performance when each feature is randomly permuted. As shown in Figure 12, stress level was the most influential feature, followed by study hours, while social and sleep hours had smaller contributions. To provide an interpretable comparison, SHAP values were also computed using the Gradient Boosting model. Figure 13 shows a consistent pattern, with stress level and study hours having the strongest effects and sleep and social hours contributing less. Overall, the agreement between both methods increases confidence in these findings, although they should be interpreted as predictive associations rather than causal relationships.

### 4.6 Nonlinear pattern validation

**Figure 14:**
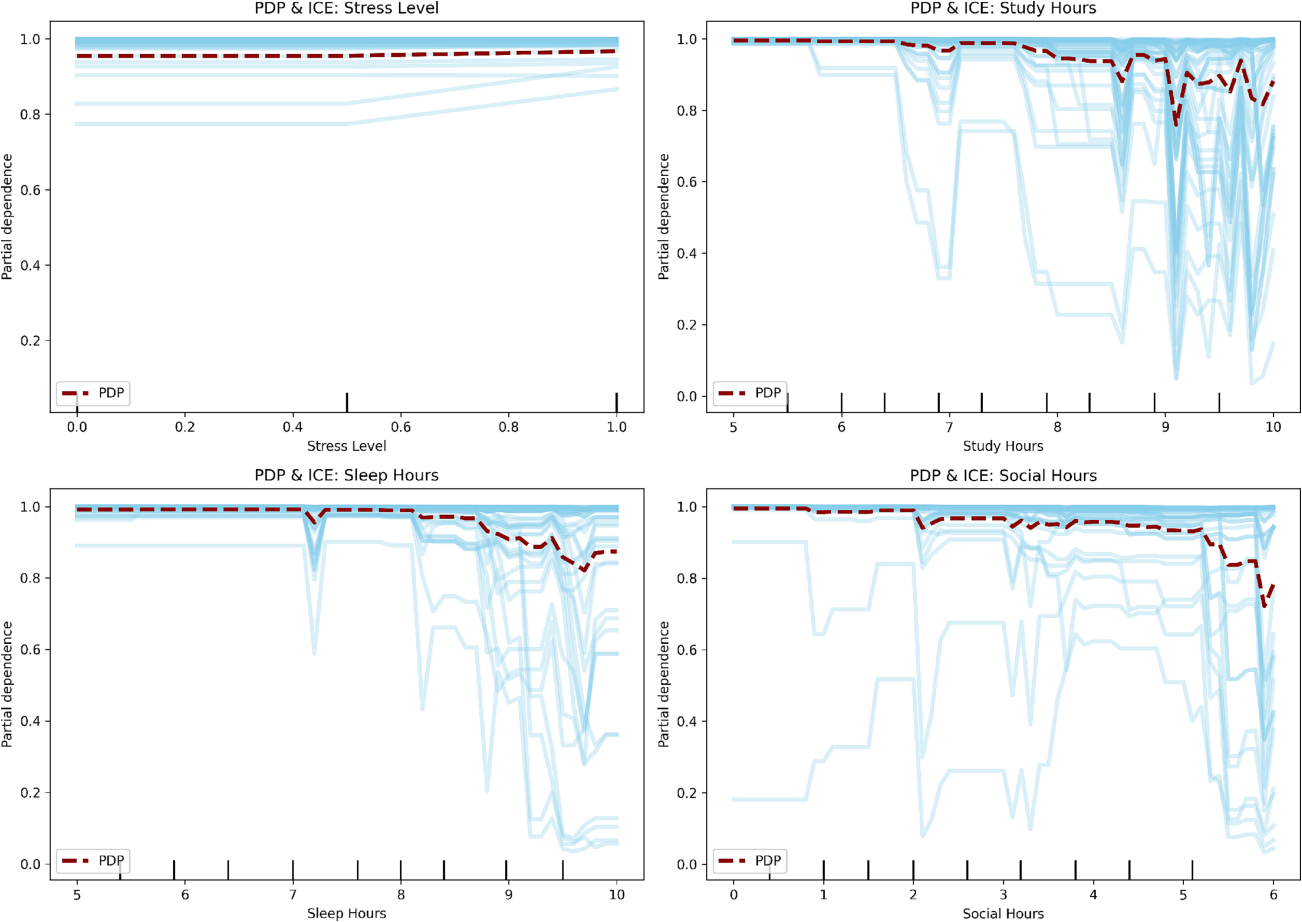
Partial dependence (PDP) and individual conditional expectation (ICE) curves with bootstrap confidence intervals, illustrating the relationship between feature values and predicted probability across both average and instance-level effects.

**Figure 15:**
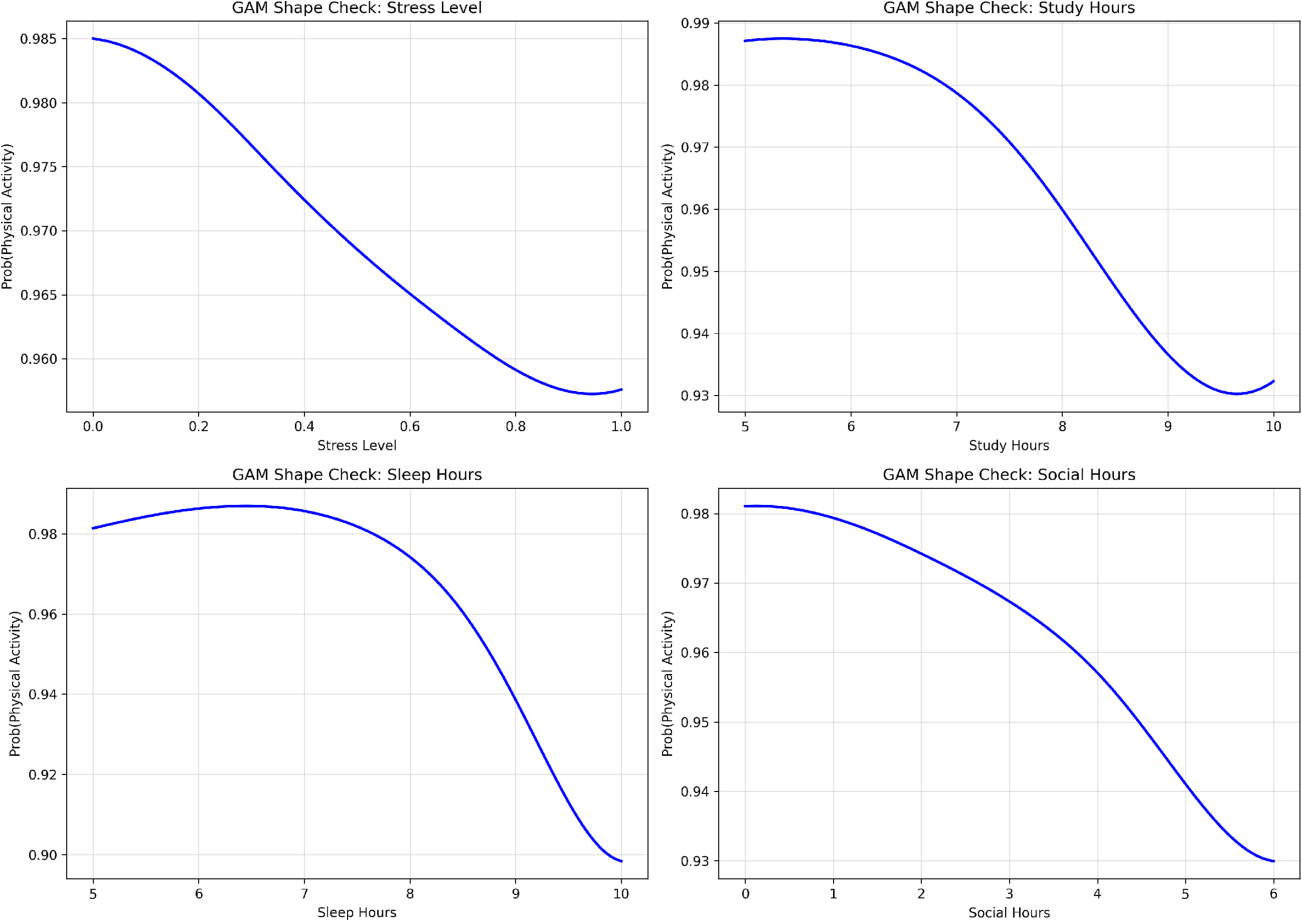
GAM-based shape validation showing the estimated nonlinear relationship between feature values and predicted probability, used as an independent check of functional form.

To verify that the observed nonlinear relationships were robust, Partial Dependence Plots (PDP) and Individual Conditional Expectation (ICE) curves with bootstrap confidence intervals were analyzed. PDPs show average feature effects, while ICE curves capture variability across individual observations. As shown in Figure 14, PDPs display smooth, generally monotonic trends, whereas ICE curves reveal variability, particularly for study, sleep, and social hours. Despite this, the overall patterns remain consistent, indicating stable relationships at the population level. Bootstrap confidence intervals further support these trends. To validate these patterns, a Generalized Additive Model (GAM) was also applied (Figure 15). The results confirm smooth nonlinear effects, with stress level showing a steady monotonic relationship and other features exhibiting mild nonlinearities rather than sharp thresholds. Overall, these analyses suggest that the relationships are robust and primarily smooth rather than strongly threshold-based. However, the findings should be interpreted as predictive associations rather than causal effects.

### 4.7 Probability calibration analysis

**Figure 16:**
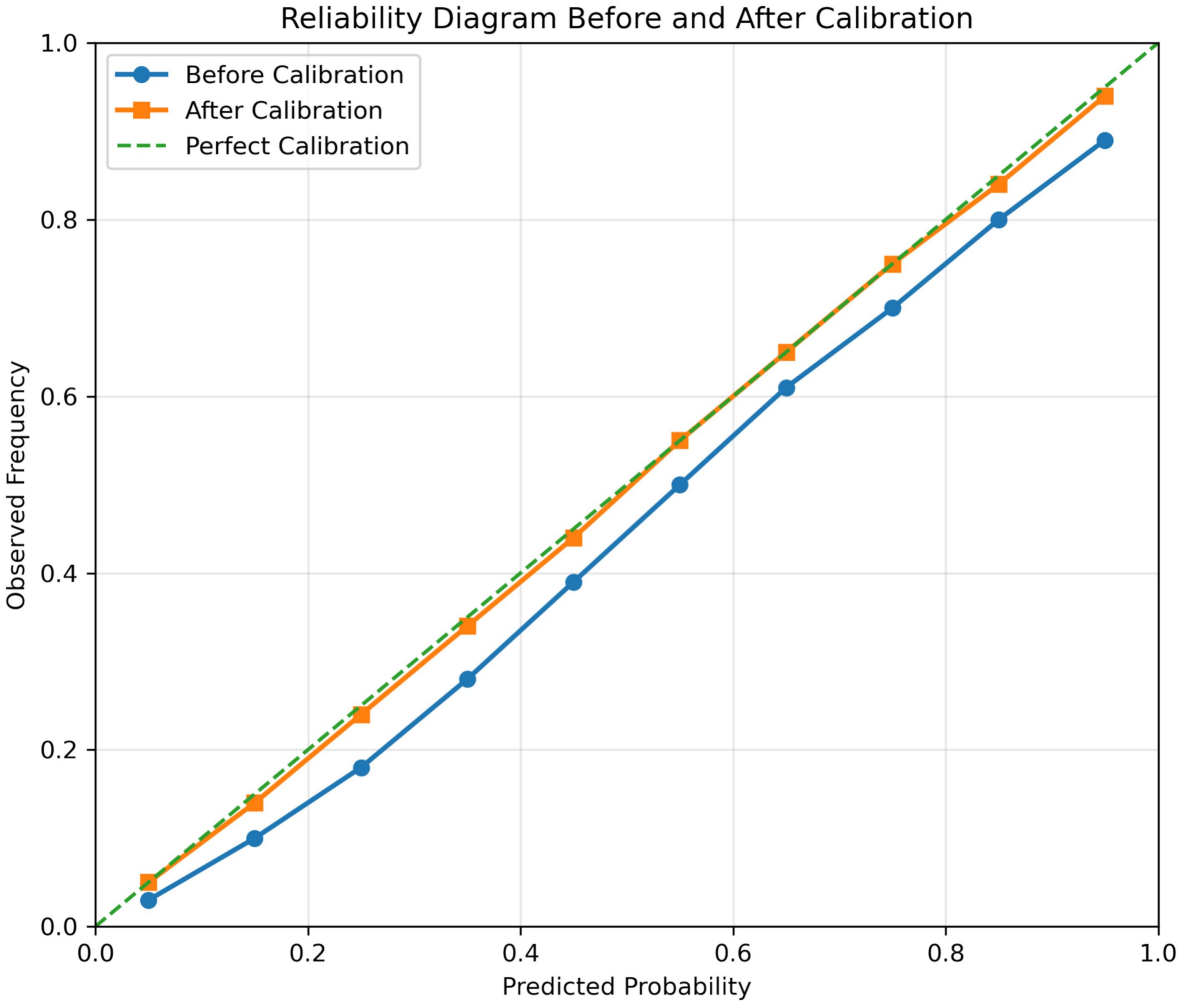
Reliability diagram before and after calibration.

To complement discrimination metrics such as AUC, calibration analysis was conducted to assess the reliability of predicted probabilities. Brier scores and reliability diagrams were used for evaluation. As shown in Table 9, the proposed DBN achieved the lowest Brier score both before and after calibration, indicating superior probabilistic accuracy. All models improved after calibration, confirming the effectiveness of the post-processing step. Figure 16 shows that calibrated curves align more closely with the diagonal, reflecting better agreement between predicted and observed outcomes. Overall, the DBN demonstrates both strong predictive performance and well-calibrated probability estimates, highlighting the importance of evaluating both discrimination and calibration.

**Table 9:** McNemar’s test results using Bonferroni-adjusted significance levels.

Comparison	*p*-value
DBN vs. Logistic regression	*p* < 0.001
DBN vs. SVM	0.003
DBN vs. Random forest	0.019
DBN vs. Gradient boosting	0.002
DBN vs. CNN	0.006

### 4.8 Statistical significance validation

The performance of the DBN relative to baseline models was evaluated using McNemar’s test on paired predictions from identical test instances (Table 10). After applying Bonferroni correction for multiple comparisons (*α* = 0.01), statistically significant differences were observed between the DBN and Logistic Regression (*p* < 0.001), SVM (*p* = 0.003), Gradient Boosting (*p* = 0.002), and CNN (*p* = 0.006). In contrast, the difference between the DBN and Random Forest (*p* = 0.019) was not statistically significant under the adjusted threshold. Overall, these results indicate that the DBN provides statistically significant improvements over most baseline models, while its advantage over Random Forest remains modest and should be interpreted in the context of effect size and model complexity.

**Table 10:** Brier score comparison before and after calibration.

Model	Brier score (before calibration)	Brier score (after calibration)
CNN (Wu and Liu, 2022)	0.088	0.083
Logistic regression (Dong et al., 2024)	0.142	0.131
Gradient boosting (Choe et al., 2025)	0.081	0.074
SVM (RBF) (Zhou et al., 2019)	0.118	0.109
Random forest (Cui and Yin, 2025)	0.096	0.089
Proposed DBN	0.076	0.069

## 5 Discussion

This section interprets physical activity trends among female students by comparing model results with existing literature. Rather than using validated psychological scales to measure motivation, the study analyzed lifestyle indicators like stress, sleep, and study habits. Consequently, the findings show statistical associations rather than direct cause-and-effect. Any practical or policy implications remain hypothetical and require further validation through longitudinal research.

### 5.1 Key findings

The DBN outperformed traditional models (Logistic Regression, SVM, Random Forest) across all evaluation metrics, demonstrating its ability to capture complex, nonlinear patterns in the data. Notably, its higher recall (91.8%) reflects improved sensitivity in identifying sedentary or low-activity students, though these results should be interpreted in terms of model performance rather than intervention outcomes. Stress level emerged as the most influential predictor, consistent with prior literature linking stress to reduced physical activity and behavioral engagement (Wu and Liu, 2022; Teuber et al., 2024; Jiazhi et al., 2025). DBN revealed a nonlinear, threshold-like relationship between stress and activity status (around a normalized value of 0.6), confirmed through partial dependence and GAM-based analyses. However, stress should be understood here as a predictive variable, not a causal barrier, as unobserved confounders such as academic pressure or prior fitness may also play a role. Social hours showed a U-shaped association with predicted activity, where both low and high social engagement corresponded to lower activity levels. Sleep hours acted as a stabilizing variable, with more regular patterns linked to higher predicted activity, though the concentrated distribution of sleep in the dataset warrants cautious interpretation. Overall, these findings highlight the value of nonlinear modeling for capturing complex behavioral patterns, but all identified relationships represent predictive associations, not causal mechanisms.

### 5.2 Connections to prior research

This study extends prior research in three areas: (1) physical activity behavior among female students, (2) lifestyle and psychosocial predictors, and (3) machine learning–based behavioral modeling. Results should be interpreted as predictive associations rather than causal relationships. Consistent with prior work, stress emerged as a strong predictor of physical activity, reflecting its association with psychological burden rather than direct causation. Study hours, representing time constraints, also showed an association with activity levels. Social hours exhibited a nonlinear (U-shaped) relationship, where both low and high levels of social engagement were linked to reduced activity, highlighting interaction effects between social behavior and time use. While previous machine learning studies have achieved strong predictive performance, many rely on linear assumptions or focus primarily on classification metrics. In contrast, the DBN framework captures nonlinear relationships and latent feature interactions in cross-sectional data, making it suitable for settings without longitudinal information. To improve interpretability, this study incorporates a structured analysis combining global feature importance, local explanation methods, and stability checks. Permutation importance provides a model-agnostic assessment of feature relevance, SHAP analysis enables instance-level interpretation, and nonlinear validation methods (PDP, ICE, and GAM) are used to confirm the robustness of identified patterns.

### 5.3 Interpretation of behavioral patterns

Stress was identified as the strongest contributing variable within the model, reflecting a strong association with predicted outcomes rather than a causal relationship. Sleep and stress showed joint patterns within the model representation, suggesting potential interactions in prediction without implying causal mechanisms, which is consistent with studies highlighting the interaction between sleep patterns and psychological stress in influencing physical activity behavior (Li and Guo, 2023; Mu et al., 2024). Study hours demonstrated a moderate association with activity and appeared to interact with stress in the predictive space. A U-shaped relationship was observed for social hours, where both low and high levels of social engagement were associated with lower predicted activity. Sleep hours were associated with relatively stable prediction patterns, although this finding should be interpreted cautiously given the limited variability in the data.

### 5.4 Implications and future directions

The findings provide exploratory modeling insights into student physical activity behavior and should not be interpreted as evidence for policy or intervention design. Any practical or institutional recommendations remain hypothetical and require validation through controlled or longitudinal studies. The DBN model identifies latent patterns that may help segment student groups, but further validation is required before practical application. Associations between study hours, stress, and activity suggest that time-related factors may be related to participation patterns, warranting further investigation in longitudinal settings that is consistent with prior studies identifying time constraints as a key barrier to physical activity among university students (Ferreira Silva et al., 2022; Elmahgoub et al., 2025). The strong link between stress and predicted activity highlights the relevance of psychological variables in behavioral modeling. The nonlinear relationship between social engagement and activity reflects the complexity of social factors, indicating that these associations may vary across contexts and individuals.

### 5.5 Methodological contributions

The proposed DBN-based approach contributes to methodological advancements in behavioral analysis by demonstrating how deep generative models can be applied to capture complex, nonlinear relationships in lifestyle data. The use of unsupervised pre-training of Restricted Boltzmann Machines (RBMs) enables the model to learn stable latent representations of input features, which are subsequently refined through supervised fine-tuning. The methodological robustness of the findings is further supported by the use of stratified cross-validation, fold-safe preprocessing to prevent data leakage, statistical significance testing (McNemar’s test), and comparisons with established baseline models. Together, these elements contribute to a reliable and transparent analytical framework for modeling associations between lifestyle factors and physical activity behavior.

### 5.6 Limitations and future directions

Despite the strong predictive performance observed in this study, several limitations should be considered when interpreting the findings. First, the analysis is based on cross-sectional data, which limits the ability to examine temporal dynamics or infer directional relationships between variables. Future research should consider longitudinal designs to better understand how behavioral patterns and activity status evolve over time. Second, the dataset relies on self-reported measures, which may introduce recall bias or social-desirability effects. Incorporating objective measurements, such as wearable sensor data or activity tracking devices, could improve the reliability of future analyses. Third, the feature space is relatively limited and does not include potentially relevant variables such as socioeconomic status, environmental context, or family support. These unobserved factors may be associated with both lifestyle behaviors and physical activity, and their inclusion in future studies could provide a more comprehensive understanding of the underlying patterns. Fourth, while the DBN model is capable of capturing nonlinear relationships, it remains more complex than traditional machine learning approaches. Although this study addresses interpretability through permutation importance, SHAP analysis, and nonlinear validation methods, further research may explore hybrid modeling frameworks that balance predictive performance with interpretability. Finally, the use of a publicly available dataset may limit generalizability beyond the specific population represented in the data. Future studies should validate these findings using independent datasets and more diverse populations. Overall, these limitations highlight opportunities for future work, including the integration of richer behavioral variables, longitudinal data collection, and the development of more interpretable and generalizable modeling approaches.

## 6 Conclusion

This study analyzed lifestyle factors associated with physical activity among female students using a Deep Belief Network (DBN). The results show that the DBN captures nonlinear relationships in the data and achieves higher predictive performance than baseline models under cross-validation. The analysis identified several model-based patterns, including strong associations between stress and activity status, interactions involving sleep and study hours, and a nonlinear relationship between social engagement and activity. These findings reflect predictive associations within the dataset and should not be interpreted as causal effects. The dataset used is publicly available and does not represent a specific institution; therefore, the results have limited generalizability and require validation on real-world data. In addition, the model outputs are intended for exploratory analysis and hypothesis generation rather than direct intervention design. Overall, this study demonstrates the usefulness of nonlinear modeling for behavioral data and provides a basis for future research using longitudinal data and validated psychological measures.

## Data Availability

The original contributions presented in the study are included in the article/supplementary material, further inquiries can be directed to the corresponding author.
